# Adsorptive Stripping Voltammetry—A New Electroanalytical Avenue for Trace Analysis

**DOI:** 10.6028/jres.093.130

**Published:** 1988-06-01

**Authors:** Joseph Wang

**Affiliations:** Department of Chemistry, New Mexico State University, Las Cruces, NM 88003

Stripping voltammetry is a powerful electroanalytical technique for trace metal measurements [1]. However, conventional stripping measurements are limited to about 25 metals that electrolytically deposit and/or form an amalgam with mercury. Hence, alternative preconcentration schemes, based on nonfaradaic processes, are desired for extending the scope of stripping voltammetry toward additional analytes.

An extremely useful, sensitive and versatile preconcentration scheme can be achieved via controlled interfacial accumulation of the analyte onto the surface of the working electrode [2]. The voltammetric response of the surface-confined species is directly related to its surface concentration, with the adsorption isotherm providing the relationship between the surface and bulk concentrations of the adsorbate. The most frequently used isotherm is that of Langmuir.

The surface-active characteristics of numerous organic analytes (that commonly complicate their conventional voltammetric measurements) can be exploited for obtaining effective adsorptive accumulation. Trace levels of reducible and oxidizable compounds, such as cardiac glycosides, tetracyclines, phenothiazines, riboflavin, streptomycin, bilirubin, diazepam, tricyclic antidepressants, or mitomycin C, can thus be determined at mercury and carbon electrodes. (Using carbon paste electrodes, both adsorption and extraction occur simultaneously.) Figure 1 illustrates the inherent sensitivity of differential pulse adsorptive stripping voltammetry, as applied to measurement of 5 × 10^−9^ mol/L digoxin. In addition to low molecular weight compounds, large biological macromolecules, e.g., cytochrome C, chlorophyll, ferritin, or DNA can also be measured. In such cases, the adsorptive approach results not only in enhanced sensitivity, but also a more favorable interaction between the electrode and the redox center of the molecule (due to conformational changes), and hence with enhanced reversibility.

In addition to organic analytes, the formation and interfacial accumulation of appropriate surface-active metal complexes onto the hanging mercury drop electrode permit trace measurement of additional metals. Figure 2 illustrates the steps involved in such measurements of metal ions. In particular, the unique voltammetric and interfacial behaviors of metal chelates of dihydroxyazo dyes allow convenient trace measurement of titanium, thorium, aluminum, iron, uranium, manganese, yttrium, or gallium. Other chelators, e.g., dimethylglyoxime, catechol, oxine, tropolone, or cresolphthalexon, are useful for trace measurements of nickel, vanadium, molybdenum, tin or lanthanum, respectively. Overall, this activity resulted in procedures for measuring more than 25 trace metals; coupled with conventional stripping schemes, about 45 elements are now measurable by stripping analysis. Simultaneous measurement of 2–3 metals is possible, based on an appropriate separation of the metal-chelate peak potentials. Because of its fundamentally different detection principles, the metal chelate approach provides different spéciation information compared to conventional stripping measurements, with the fraction of metal measured including the free ion and metals displaced from natural complexes during the formation of the strong adsorbable chelate. When the chelating ligand is present in large concentration excess compared to natural ligands, and a very strong chelate is formed, the *total* metal content is determined. The different nature of the response results in improved performance for metals, e.g., tin or gallium, measurable also by conventional stripping schemes, because interferences (e.g., overlapping peaks, intermetallic compounds) are minimized. Short preconcentration periods result in detection limits as low as 10^−10^–10^−11^ mol/L. Lower levels, e.g., 10^−12^ mol/L platinum, can be achieved upon coupling with catalytic reactions (i.e., controlled adsorptive accumulation of the catalyst). Such dual amplification is expected to play an increasing role for ultratrace measurements of species exhibiting adsorption-dependent hydrogen catalytic processes. At low analyte levels (10^−7^–10^−10^ mol/L), for which the method is usually applied, a linear adsorption isotherm is obeyed and the response is linear. New strategies, such as the use of permselective electrode coatings or the medium-exchange approach, result in substantial improvements in the selectivity and reproducibility. For example, the medium-exchange approach allows convenient measurement of dopamine in the presence of large excess of ascorbic acid. Interferences due to coadsorbing surfactants can be minimized by covering the electrode with a cellulose acetate film.

Adsorptive stripping voltammetry is now a highly sensitive and rapid technique, applicable to analyses in various fields. Its utilization is expanding rapidly and will continue to do so in the near future.

## Figures and Tables

**Figure 1:**
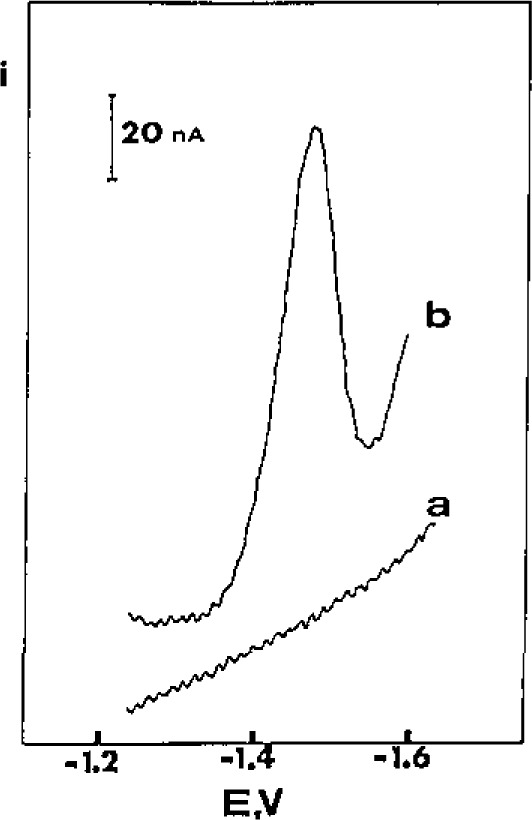
Voltammograms for 5 nmol/L digoxin using (a) no and (b) 15-minute accumulation.

**Figure 2:**
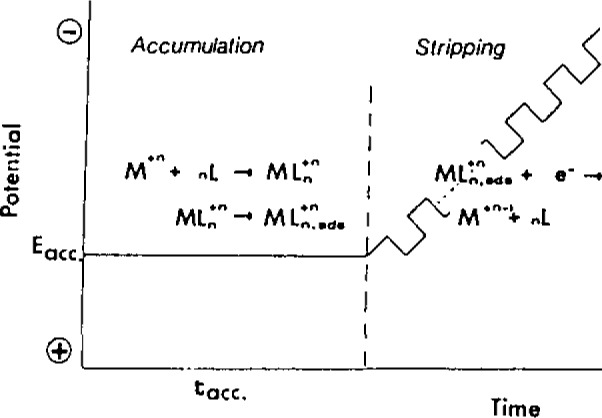
Steps in adsorptive stripping measurements of a metal ion based upon the formation, accumulation and reduction of its surface active complex.
